# The cytological analysis of crossing over in armadillos supports the existence of a phylogenetic component of recombination rates in mammals

**DOI:** 10.1371/journal.pone.0326703

**Published:** 2025-06-26

**Authors:** Luis Francisco Rossi, María Inés Pigozzi

**Affiliations:** Instituto de Investigaciones Biomédicas, INBIOMED (Universidad de Buenos Aires- CONICET), Buenos Aires, Argentina.; Centre de Recherche en Biologie cellulaire de Montpellier, FRANCE

## Abstract

In mammals rates of recombination are well predicted by the phylogenetic relationship between species, with lower recombination rates in more basal clades. In this regard, there is currently insufficient evidence for Xenarthra, one of the earliest branches of eutherian mammals. Here we estimated the average recombination rates in four species of armadillos (Cingulata, Xenarthra) using immunodetection of the protein MLH1, a reporter of reciprocal recombination, in pachytene. The recombination rates of the examined species are strikingly similar; despite the fact that they split more than 40 million years ago, suggesting that this may be a conserved trait in other Xenarthra. We provide evidence that armadillos have lower recombination rates than the average for eutherian mammals, and that they approach those of rodents more closely than those of early mammalian clades like Afrotheria.

## Introduction

During the first meiotic division, recombination promotes genetic variation in haploid genotypes and creates physical linkages between homologs, hence helping to secure chromosomal segregation. Even though balanced segregation during meiosis I only requires one crossover per chromosome, the number of homologous recombination events exhibits significant quantitative variation above this minimum within and between species. A consequence is that recombination rates, that is, how frequently crossovers occur during meiosis, are far from being conserved, varying substantially between species, populations, individuals and, sometimes between sexes of the same species. It has been suggested that the variation in recombination rates may contribute to patterns of biological diversification, but whether this fluctuation is adaptive or neutral, and what factors might shape it, remain unclear [1]. Due to their significant implications for evolution and population genetics, there has been considerable effort to understand how and why recombination rates diverge [2,3]. The estimates of how frequently meiotic recombination occurs in a genome are commonly expressed as the recombination frequency per mega- or kilobase per generation and can be measured at different genomic resolutions. The recombination frequency and landscape can be determined with great accuracy thanks to advancements in high-throughput sequencing and genotyping technology. When applied to population level, these methods yield estimates of historical recombination, whereas linkage maps offer an estimate of crossover events that occurred over a few generations [3,4]. The results obtained with these approaches rely on the density of markers or the genome’s sequencing coverage. Since these features often differ significantly across analyses, comparisons between different studies can be difficult. Genome-wide recombination can also be measured using cytological methods, where crossover events are directly observed in germ cells during diplotene/diakinesis. Cytological methods are particularly suitable for measuring the genome-wide recombination rate in wild individuals since, unlike linkage maps, they do not require crosses or the identification of relatives. A valuable direct approach is the immunodetection of the recombination protein MLH1 (MutL-homolog 1), which forms foci at the sites of successful crossover events due pachytene [5,6]. MLH1-focus maps in pachytene spreads are now common in the literature and have been generated for many species [7,8]. They can provide useful data to detect large-scale differences in crossing over between chromosomes, chromosome arms, or chromosome segments. In addition, MLH1 maps share common procedures and measurement units that allow for comparative inference between studies and across larger taxonomic scales [9,10].

**Fig 1:**
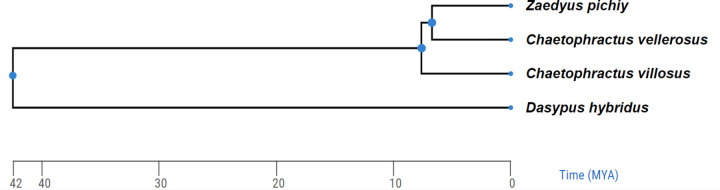
Cladogram of the analyzed species. The tree was obtained from TimeTree (Kumar et al 2017).

In mammals, linkage studies gave insights into the range of RR variation, showing that the human genome had twice as much recombination as a mouse’s, and it was several times higher compared to marsupials [11–13]. At kb level, recombination rates appear to evolve rapidly between closely related species, but their evolution on the broader scale is less understood [3,14]. Comparative analyses of global rates from both linkage and MLH1-focus maps have suggested that the mammalian species that diversified earlier in the evolutionary tree have lower recombination rates than those from more derived phylogenetic branches [15,16]. Despite the fact that these analyses cover several mammalian evolutionary lineages, certain taxa, including Xenarthra, were not represented. The superorder Xenarthra is one of the four recognized major eutherian clades alongside Afrotheria, Euarchontoglires and Laurasiatheria [17,18]. A comprehensive phylogenetic analysis situates the divergence within Atlantogenata (Afrotheria and Xenarthra) in the Cretaceous Period more than 90 Mya [19]. A number of large, time-scaled phylogenies show that the mean divergence times of Xenarthra occurred about 70 million years ago (Mya), with their main ordinal diversification of occurring during the Paleocene [19–22]. Extant Xenarthra are currently represented by 39 species grouped in 14 genera that include the armadillos, the anteaters and sloths [23]. The armadillos are grouped in the order Cingulata that diverged from Pilosa (sloths and anteaters) about 42.5 Mya (range 33.0–52.6 Mya) [24,25]. Given the basal position of Xenarthra among placental mammals, we used armadillos as super-ordinal representatives to examine the relationship between recombination rates and phylogeny, focusing on the reciprocal exchange of genetic information between homologous chromosomes during meiosis. Taking into account the relationship reported between phylogeny and recombination rates in eutherians, armadillos should have, on average, lower crossover numbers than more recently diverged mammalian lineages. To test this prediction, recombination rates were obtained counting the number of MLH1 foci in spermatocytes at the pachytene stage in four species of armadillos from the families Chamyphoridae and Dasypodidae (Fig 1). We discovered that the genome-wide recombination rates are very similar and that these rates are lower compared to other groups of mammals that have undergone more recent divergence, such as primates, carnivorans, or artiodactyls.

## Materials and methods

### Specimens

Testis samples were collected from animals that were severely injured in road accidents, or that were captured in the field as part of more comprehensive investigations of reproductive aspects of these species. The sampling locations and number of specimens used in the present study are detailed in the S1 File. This study was carried out in compliance with the guidelines for the use of wild mammals in research and experimental procedures of the AMVA (https://www.avma.org/sites/default/files/2020-02/Guidelines-on-Euthanasia-2020.pdf). The protocol was approved by the Institutional Animal Care and Use Committee of the School of Medicine, Universidad de Buenos Aires (Permit # EX-2023–02333068- -UBA-DMEA#FMED). All efforts were made to minimize suffering during the handling of the specimens.

### Synaptonemal complex spreading and immunostaining

We prepared synaptonemal complex spreads from the freshly isolated testes using a drying-down method for mammalian spermatocytes [26] followed by immunostaining with a standard protocol for meiotic cell staining [27]. Briefly, the slides were incubated overnight at 37ºC in a humid chamber with the following primary antibodies: a rabbit polyclonal anti-SYCP3 (1:500; Abcam, Cambridge, UK), mouse monoclonal anti-MLH1 (1:30; BD Biosciences, USA) and human anti-centromere (1:50; Laboratorio IFI, Buenos Aires, Argentina). Fluorochrome-conjugated secondary antibodies were used for detection (Jackson ImmunoResearch Laboratories, PA, USA), incubating for one hour at room temperature. Preparations were counterstained with DAPI, mounted in a glycerol-based media with an antifading agent and visualized using a Leica epifluorescence microscope equipped with the appropriate filters. Separate pictures were taken for each fluorochrome using a charged coupled device camera (Leica DFC 300 FX), and then overlapped in Adobe Photoshop CS5.

### Image analysis and statistics

Foci were scored in each nucleus using the Count tool of Adobe Photoshop. After compiling the focus data, the total length of the genetic map results from multiplying the average number of MLH1 foci in each species multiplied by 50 cM. The recombination rates in cM/Mb were calculated dividing the genetic map by the genome size expressed in Mbp. Statistical calculations and graphs were done using Graph Pad Prims 8.

### Mammalian recombination database

A database of chiasmata, MLH1 foci and linkage maps in mammals [28] was used to calculate average recombination rates in 83 species from 15 mammalian orders (S2 File). The recombination rates (cM/Mb) were calculated by multiplying the number of chiasmata (or MLH1 foci) by 50 to obtain the cM map length and then dividing by the genome size in Mbp. Genome size estimates were obtained from the Animal Genome Database [29], from published literature or from other online sources indicated in the S2 File. When genome sizes were expressed in pg, they were converted to Mbp using a factor of 978 [30]. Recombination rates derived from linkage maps were only included where deep coverage was known in order to reduce under- or overestimation. The recombination rates obtained for armadillos in the present work were incorporated into the database.

## Results and discussion

### MLH1 foci counts give insights of crossover requirements during mammalian meiosis

**Fig 2:**
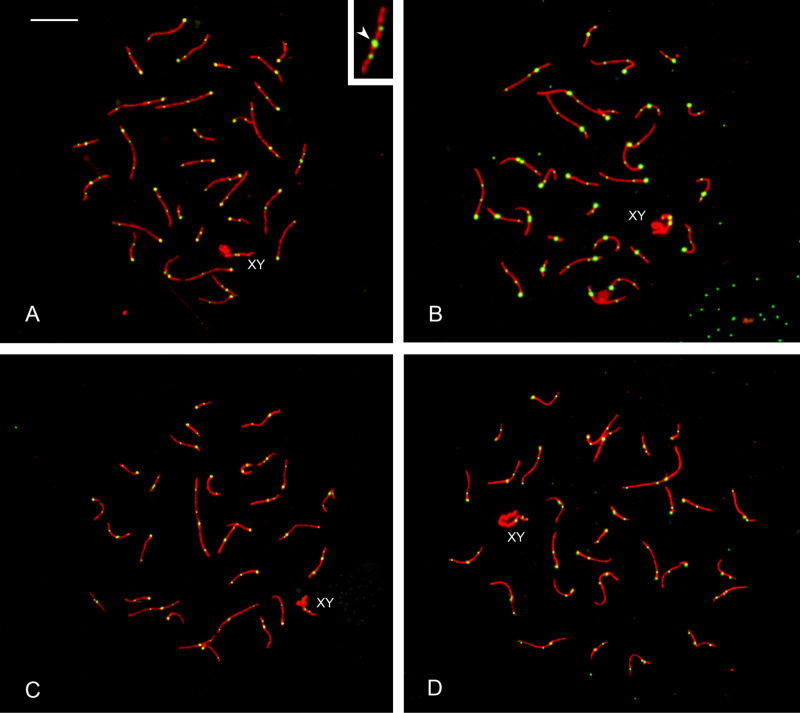
Representative images of pachytene spermatocytes. A) *D. hybridus*, B) *C. villosus*, C) *C. vellerosus* and D) *Z. pichiy*. Cells were immunostained for the mismatch repair protein MLH1 (green foci), which localizes to sites of crossing over; the synaptonemal complex protein SYCP3 (red); and for centromeric proteins (green). The morphological difference between the MLH1 foci and the centromeric signal (arrowhead) is displayed in the inset. Bar: 10 µm.

The diploid chromosome numbers in the studied species range from 60 to 64, and the bivalents found in the pachytene cells are consistent with the expected haploid set for each species. Fig 2 shows representative pachytene spermatocytes of the four species immunostained for the synaptonemal complex component SYCP3, MLH1 and centromeric proteins.

A total of 134 pachytene spermatocytes were analyzed for the number of SCs and MLH1 foci on the autosomal bivalents (S1 File). Foci were counted only on the autosomal bivalents because recombination in the XY pair of mammals follows a different dynamic of double-strand break processing during meiosis [31]. In each species the frequency of bivalents that lacked MLH1 foci was less than one percent and they were predominantly small synaptonemal complexes. These frequencies are within the limits found on studies of house mouse and may reflect the transient nature of MLH1 foci during pachytene [32]. The number of MLH1 foci was remarkably similar with only 3% variation between species (Table 1). A one-way ANOVA test revealed that the total recombination rates estimated as the mean number of MLH1 foci per cell are not significantly different between species (F_(3,130)_ = 2.468; P < 0.0650).

**Table 1:** Karyotype and MLH1 foci in armadillo spermatocytes.

Species	Karyotype		MLH1 foci				
	2n	FN/2	N of cells	Minimum	Maximum	Average	SD
*D. hybridus*	64	38	30	35	44	39.2	2.2
*C. villosus*	60	42	36	35	43	39.3	2.2
*C. vellerosus*	62	44	37	35	44	39.5	1.9
*Z. pichiy*	62	44	31	34	43	38.2	1.9

The diploid numbers (2n) are from: *D. hybridus* [33,34]; *C. villosus* [35]; *C. vellerosus* [34]; *Z. pichiy* [33]. aFN/2 is the haploid number of autosomal chromosome arms.

This similarity is rather unusual considering that the number of crossovers can vary significantly even between individuals of the same species in mammals and other organisms [1,36,37].

Individual autosomal bivalents were not related to their mitotic counterparts because there are too many overlaps in centromere locations and synaptonemal complex lengths for a reliable correlation. One exception is the largest bivalent of *C. villosus*, *C. vellerosus* and *Z. pichiy* which is metacentric with a centromeric index of 0.42 to 0.44 in pachytene spreads, in agreement with morphological data from mitotic chromosomes [34,35]. In all three species, we discovered that MLH1 foci are only found in the long arm of the bivalent. The absence of MLH1 foci may respond to the presence of heterochromatin, a recognized inhibitor of crossing over [38]. In fact, the short arm of this chromosome stains darker than the euchromatic long arm after C-banding and has poor definition after G-banding [34,35]. In *Dasypus hybridus*, there are no chromosomes or chromosome arms with similar staining, suggesting that this putative heterochromatic block is not present in Dasypodidae [34,39]. A visual examination of pachytene nuclei in *Dasypus* revealed that the largest synaptonemal complex, which is acrocentric, has two foci in most nuclei without any specific localization (Fig 2A). Both mitotic and the present meiotic observations point to a lack of conservation of the largest chromosome pair in Chlamyphoridae and Dasypodidae. Specific FISH markers along with MLH1-focus detection should be employed to determine whether broad-scale recombination rates at homologous chromosomal segments are conserved in armadillos.

**Fig 3:**
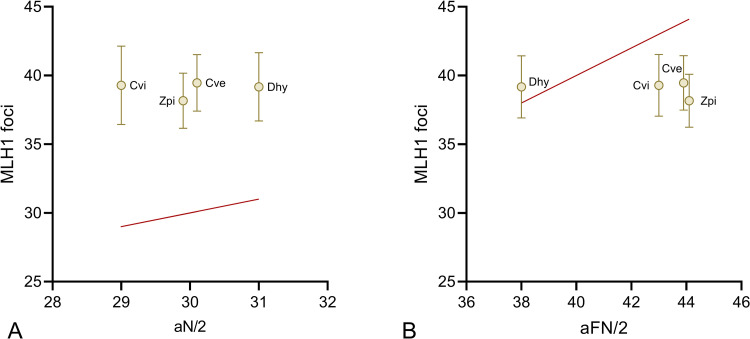
Relationship between MLH1-focus number and karyotype. Distribution of mean (± SD) MLH1 focus- counts in armadillos as a function of (A) the number of autosomes in the haploid set (aN/2) and (B) the haploid number of autosomal chromosome arms (aFN/2). The points correspond to species values for *D. hybridus* (Dhy), *C. villosus* (Cvi), *C. vellerosus* (Cve) and *Z. pichiy* (Zpi). A. All the species harbor more than one crossover per chromosome as demonstrated by the fact that all points fall above the y = x line corresponding to one crossover per chromosome. (B) Three of the examined species have, on average, fewer crossovers than chromosome arms. Only the point for *D. hybridus* fall above the solid line that represents number of crossovers = number of chromosome arms in the haploid set. The dots for the species with the same number of autosomal bivalents (A) or the same haploid autosomal number (B) are slightly shifted around 30 and 44, respectively, for visual purposes.

Among the species included in the current study, *Dasypus hybridus* has the lowest fundamental number, that is, has fewer chromosomal arms, because its karyotype comprises more acrocentric chromosome pairs. The difference provides an opportunity to investigate the relationship between the fundamental number and the amount of crossovers per bivalent. During the first meiotic division, at least one crossover per chromosome pair is required in order to ensure faithful segregation. It has been extensively argued that, among mammals, the number of chromosome arms sets the lower bound of crossover rates to accomplish this requirement [40]. More recently, extensive analysis of 112 mammalian species established that this structurally required minimum has undergone multiple independent shifts from one crossover per chromosome arm to one per chromosome during mammalian evolution [28]. As shown in Fig 3 armadillos have, on average, more MLH1 foci than autosomal bivalents, and they also have fewer average foci than haploid chromosome arms, excepting *D. hybridus*.

If the global recombination rate is constrained by a minimum of one crossover per chromosome arm, the average number of foci should be greater than or equal to the haploid number of autosomal chromosome arms [28]. This requirement is not met in armadillos because MLH1 data from the four species shows that almost 70% of the spermatocytes have fewer foci than expected under the premise of one mandatory crossover per chromosome arm (S1 File). As previously mentioned, the species analyzed here belong to the families Dasypodidae and Chlamyphoridae which diverged 42 Mya, according to morphological and molecular DNA data [25]. Since the genus *Dasypus* belongs to Dasypodidae, recognized as the most basal family among Cingulata [25], the minimum number of crossovers in the common ancestor of armadillos was first defined by the haploid number of chromosome arms and then shifted towards a less stringent requirement of one crossing over per chromosome as observed in Clamyphoridae. The present observations support the idea that the chromosomal requirement for recombination at meiosis may vary among species, and this condition has dropped below the one-per chromosome-arm threshold multiple independent times during mammalian evolution [28,41]. Given the basal position of armadillos and the relationship between crossovers and chromosome arms found here, a requirement of one crossover per chromosome arm should not be considered an ancestral character among eutherian mammals.

### Association of recombination rate and phylogeny in mammals

The rate of genome-wide recombination may vary between individuals, subspecies, populations, and domestic breeds within a given species. In the present work, we focused on the variation of contemporary recombination rates in armadillos, as representatives of Xenarthra, one of the four major clades among eutherian mammals. Average rates were calculated from the MLH1 focus frequencies and the total amount of DNA and are expressed in cM/Mb (Table 2).

**Table 2:** Genomes size and recombination rate in armadillos.

Species	Genome (pg)	Genome (Mbp)	Foci/cell	cM	cM/Mb
***D. hybridus***	4.89	4782	39.2	1960	0.41
***C. villosus***	4.18	4088	39.3	1965	0.48
***C. vellerosus***	4.46	4362	39.5	1975	0.45
***Z. pichiy***	4.21	4117	38.2	1910	0.46

The genome sizes in pg are from [42].

The average rate across the four species is 0.45 cM/Mb, with small variations between species, as would be expected given the similar MLH1 focus frequencies and the scarce differences in genome sizes. Recombination studies for anteaters and sloths are not available, making it difficult to predict the conservation of this character at the superordinal level. Despite belonging to families that split more than 40 million years ago, the species under analysis exhibit similar rates of crossing-over, as indicated by MLH1 counts. At a broad scale, genomic recombination rates evolve at a slower pace compared to recombination at a finer level [15], therefore, it is conceivable that recombination rates in armadillos can be used to predict those of other superorder members.

**Fig 4:**
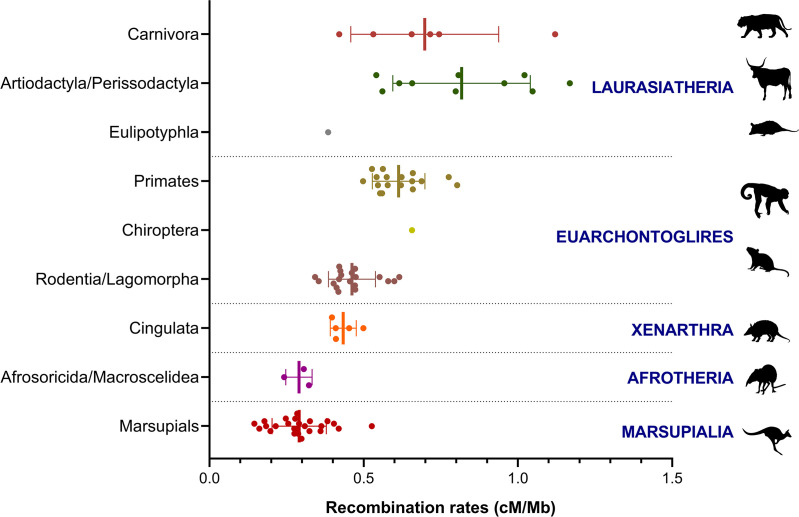
Recombination rates in mammals. Summary representation of the recombination rates in 85 mammalian species. Eutherian representatives are grouped by orders, with the main recognized superorders listed on the right. Each dot represents a species recombination rate that was taken from the S2 File.

Previous analysis based on thirteen mammalian species from five orders established a strong phylogenetic effect in the crossover numbers [15]. In order to provide a more comprehensive view of global recombination rates in mammals, we collected the genomic sizes and recombination data for 85 species, including the representatives of Xenarthra studied here. (Fig 4; S2 File).

Our present analysis shows that Marsupials and Afrotheria, with 0.29 cM/Mb, have the lowest recombination rates (S3 File). In armadillos the recombination rates (0.45 cM/Mb) are closer to those in Rodentia and Eulipotyphla (*Sorex araneus*), while in Laurasiatheria (Artiodactyla and Carnivora) most species have recombination rates above the average for eutherian mammals. When the recombination rates are plotted against the average divergence times of each group, a strong correlation is observed (Spearman´s r = 0.9167; P: 0.0001; S3 File). If the quantity of recombination per se (measured in cM) is taken into account alone, the trend also holds, indicating that lower recombination rates are not caused by basal mammals’ larger genomes (Spearman´s r = 0.7000; P: 0.0433; S3 File). However, this correlation is sensitive to the removal either of the extreme values (Marsupials or Artiodactyla), suggesting the phylogenetic signal in the mammalian recombination rates may be at least partially explained by the variation in genome size across the same phylogeny.

## Supporting information

S1 File MLH1-focus counts per species. (XLSX)

S2 File Database of global recombination rates in mammals. (XLSX)

S3 File Divergence time and recombination in mammals. (PDF)
