# Supplementary material for: The cytological analysis of crossing over in armadillos supports the existence of a phylogenetic component of recombination rates in mammals
Source: PLoS One. 2025 Jun 26;20(6):e0326703. doi: 10.1371/journal.pone.0326703 (PMC12200783; doi:10.1371/journal.pone.0326703)
Supplement: S3 File — (PDF) [file pone.0326703.s003.pdf]

Table S1. Divergence time and recombination in mammals

	# of species	DT (average)	Recombination rate (cM/Mb)	Genetic map (cM)
**MARSUPIALIA**	23	81	0.2908	1067
**AFROTHERIA**	3	80	0.2902	1312
**Euliphotyphla**	1	74.3	0.3848	1095
**XENARTHRA**	5	67.4	0.4326	1941
**Glires (Rodentia/Lagomorpha)**	19	61	0.4619	1318
**Primates**	17	67.1	0.6161	2032
**Chiroptera**	1	57.1	0.6565	1650
**Carnivora**	6	40.2	0.698	1992
**Artiodactyla / Perissodactyla**	10	58.4	0.8173	2540

Divergence times (DT) are from Upham et al 2019. Recombination rates are the average for each mammalian group (superorders and orders).

1. Upham NS, Esselstyn JA, Jetz W. Inferring the mammal tree: Species-level sets of phylogenies for questions in ecology, evolution, and conservation. PLoS Biol. 2019; 17(12):e3000494. doi: 10.1371/journal.pbio.3000494.

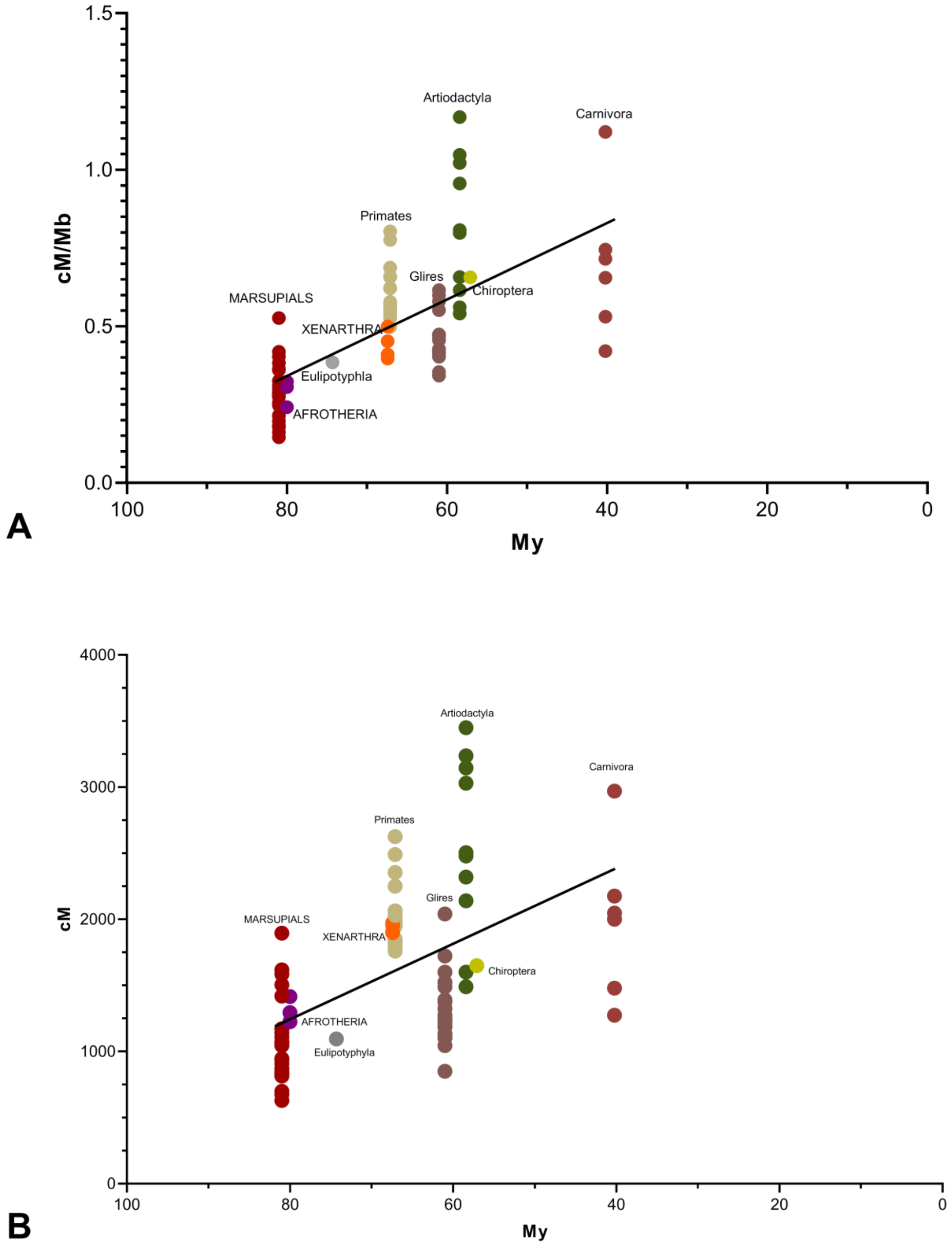

Fig S1. Correlation between divergence times and recombination in 85 mammalian species. Data on recombination rates (A) and map length (B) were extracted from S2_file. Recombination rates are lower in deeper lineages in the mammalian phylogeny than in more recently divergent species (Spearman´s r= 0.9167; P: 0.0001). This correlation is maintained even is the genome size is not taken into account (Spearman´s r= 0.7000; P: 0.0433).
